# The importance of prototype similarity for physical activity: Cross‐sectional and longitudinal associations in a large sample of young adolescents

**DOI:** 10.1111/bjhp.12582

**Published:** 2022-02-03

**Authors:** Catherine Wheatley, Thomas M. Wassenaar, Nick Beale, Piergiorgio Salvan, Helen Dawes, Emma Davies, Heidi Johansen‐Berg

**Affiliations:** ^1^ 6396 Nuffield Department of Clinical Neurosciences Wellcome Centre for Integrative Neuroimaging John Radcliffe Hospital University of Oxford UK; ^2^ Department of Sport and Health Sciences Oxford Institute of Nursing Midwifery & Allied Health Research Oxford Brookes University UK; ^3^ College of Medicine and Health University of Exeter UK; ^4^ Centre for Psychological Research Faculty of Health and Life Sciences Oxford Brookes University UK

**Keywords:** Theory of Planned Behaviour, Prototype Willingness Model, physical activity, adolescent, behaviour‐change

## Abstract

**Objectives:**

Physical activity declines during adolescence. The Theory of Planned Behaviour (TPB) is a useful framework for investigating activity but leaves variance unexplained. We explored the utility of a dual‐process approach using the TPB and the Prototype Willingness Model (PWM) to investigate correlates of physical activity, and 1‐year change in physical activity, among a large sample of adolescents.

**Design:**

A cross‐sectional and longitudinal analysis of baseline and follow‐up data from the Fit to Study cluster‐randomized trial.

**Methods:**

A total of 9,699 secondary school pupils at baseline and 4,632 at follow‐up (mean age = 12.5 years) completed measures of past week physical activity and constructs from both behaviour‐change models, at time‐points 1 year apart. Cross‐sectional analyses used multilevel, stepwise regression models to measure the strength of associations between model constructs and physical activity, and variance in behaviour explained by PWM over and above TPB. In longitudinal analyses, change scores were calculated by subtracting follow‐up from baseline scores. Models controlling for trial treatment status measured the strength of associations between change scores, and variance explained.

**Results:**

At baseline, after controlling for past behaviour, physically active prototype similarity had the strongest relationship with activity after the intention to be active. Change in prototype similarity had the strongest relationship with change in activity after the change in intention and attitudes. Prototype perceptions and willingness explained additional variance in behaviour.

**Conclusion:**

A dual‐process model incorporating prototype perceptions could more usefully predict physical activity than models based on rational expectations alone. Behaviour‐change interventions promoting an active self‐image could be tested for effects on physical activity.

**Statement of contribution**

***What is already known on the subject?***

- Physical activity declines during adolescence, a period of rapid psychosocial change.
- The Prototype Willingness Model predicts health‐risk behaviours including smoking and drinking alcohol during this developmental stage.
- Our understanding of relationships between prototype perceptions and adolescent physical activity is limited.

***What this study adds?***

- This is the largest study to investigate the predictive utility of the Prototype Willingness Model for adolescent physical activity.
- Identifies that prototype similarity has a stronger relationship with physical activity than either attitudes or subjective norms, but not intention.
- Prototype similarity predicts physical activity over and above habitual physical activity.
- Demonstrates the temporal instability of PWM and TPB variables, and that 1‐year change in prototype similarity is linked to change in physical activity.

## Background

Rapid psychosocial development during early adolescence can lead to lasting changes in health behaviours, including physical activity (Dahl, Allen, Wilbrecht, & Suleiman, 2018; Inchley et al., 2016). Regular physical activity promotes physical health and protects mental well‐being (Janssen & LeBlanc, 2010), yet activity declines during childhood and adolescence, and only around a fifth of young people meet the World Health Organisation’s recommendation of an hour a day on average of moderate‐to‐vigorous physical activity, such as brisk walking, cycling, or running (Farooq et al., 2020; Guthold, Stevens, Riley, & Bull, 2020; World Health Organization, 2016).

A better understanding of the correlates and predictors of adolescent physical activity could inform interventions with the potential to guide the developmental course in a positive direction (Sallis & Owen, 1998). Identifying promising models of physical activity behaviour change is particularly important given that non‐modifiable factors including female sex and low socioeconomic status (SES) are consistently linked with lower activity levels (Sterdt, Liersch, & Walter, 2014).

### The theory of planned behaviour

The dominant behaviour‐change theories for investigating physical activity have been in the social cognitive tradition, which assumes decisions are based on rational expectations of behavioural outcomes (Rhodes, McEwan, & Rebar, 2019). One such model, the Theory of Planned Behaviour (TPB; Ajzen, 1985), has been widely used for investigating physical activity (Ajzen & Schmidt, 2020; Buchan, Ollis, Thomas, & Baker, 2012): behaviour is determined by reflective intentions, which are a product of attitudes (evaluation of a behaviour), subjective norms (perceived social pressure to perform the behaviour), and perceived behavioural control (PBC; ability to perform the behaviour), which is governed in turn by beliefs about self‐efficacy (capacity) and control (degree of autonomy to perform the behaviour; Conner & Sparks, 2005). The TPB is a useful framework for explaining (Hagger, Chatzisarantis, & Biddle, 2002) and predicting (Hamilton, van Dongen, & Hagger, 2020; McEachan, Conner, Taylor, & Lawton, 2011) physical activity behaviour, although when people form intentions to be active, these do not always lead to active behaviour: this discrepancy (noted across numerous health behaviours and age groups) has been labelled the ‘intention‐behaviour gap’ (Rhodes & Bruijn, 2013). Consequently, research has attempted to extend the TPB by exploring potential moderators of the intention‐physical activity relationship, including both reflective factors such as affective attitudes and anticipated regret, and more automatic processes such as habit and self‐regulation (Rhodes, Cox, & Sayar, 2021).

### The prototype willingness model

A more recent, promising approach to explaining and predicting physical activity is the dual‐process framework, which accounts for both reflective and automatic determinants of behaviour (Rhodes et al., 2019). The framework recognizes that behavioural decisions appear to be influenced by constructs, such as motivation, self‐regulation, habit, and automaticity, as well as by reflective intentions (Gardner, de Bruijn, & Lally, 2011).

**Figure 1:**
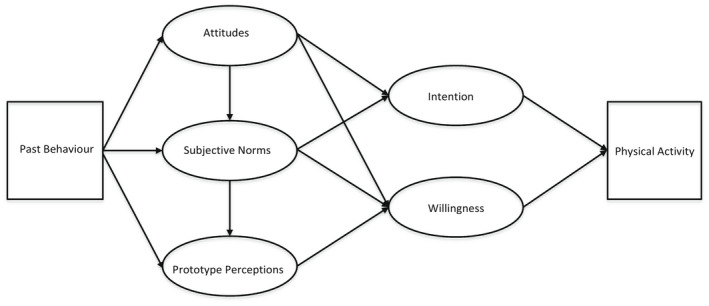
The prototype willingness model (Gerrard et al., 2008; Gibbons & Gerrard, 1995).

The Prototype Willingness Model (PWM) is a dual process approach that builds on the TPB to account for both reflective factors and the more reactive decision‐making that occurs in social situations, particularly among adolescents (Gerrard, Gibbons, Houlihan, Stock, & Pomery, 2008; Gibbons & Gerrard, 1995). The model has been widely used to explore alcohol consumption, a health‐risk behaviour that appears likely to be influenced by habit, learned associations, and social influences, as well as intentions to drink or abstain (Gibbons, Kingsbury, Gerrard, & Wills, 2011). There are two routes to behaviour. In the planned pathway, intentions, attitudes, and subjective norms determine behaviour. The pathway does not include PBC because social opportunities to engage in behaviours are said to be more influential than self‐efficacy and control beliefs in this age group (Gibbons, Houlihan, & Gerrard, 2009). In the social‐reactive pathway, behaviour is predicted by prototype perceptions and behavioural willingness. Prototypes are distinctive and widely recognized images of a certain ‘type’ of person: these can have both positive and negative characteristics. For example, young people often have a clear idea about the type of person their age that drinks, and might describe this typical person as ‘confident’ or ‘reckless’. According to the PWM, these clear and powerful images motivate behaviour through a process of social comparison. If young people find a behavioural prototype attractive or appealing (favourable), and it aligns closely with their self‐image (similar), then they are more likely to engage in the behaviour. By behaving like the prototype they will acquire its characteristics, a powerful consideration for adolescents whose self‐image is still under construction (Gibbons & Gerrard, 1995). Prototype perceptions (favourable and similar) are thought to be influenced by both peers and parents (Ouellette, Gerrard, Gibbons, & Reis‐Bergan, 1999; Ouellette, Hessling, Gibbons, Reis‐Bergan, & Gerrard, 2005). Behavioural willingness describes what individuals are open to doing in reaction to social circumstances, rather than what they plan to do (Gibbons, Gerrard, & Lane, 2003). Past behaviour is said to influence behaviour in both reasoned and reactive pathways (Figure 1).

Research exploring the utility of this dual‐process model has examined a range of health behaviours and taken two broad approaches. First, it has compared the extent to which TPB and PWM variables *explain variance* in health behaviours such as weight‐loss dieting (Instone & Davies, 2019) and speeding while driving (Elliott et al., 2017). Second, it has compared the *strength of associations* between constructs in these models with intention and willingness to engage in behaviours such as smoking and drinking (Spijkerman, van den Eijnden, Vitale, & Engels, 2004), and doping in competitive sport (Whitaker, Long, Petróczi, & Backhouse, 2014).

Two meta‐analyses have explored relationships between TPB and PWM variables and health behaviours. Todd, Kothe, Mullan, and Monds (2016) examined the relative contribution of reasoned and reactive pathways to behaviour across 90 studies. The PWM explained 20.5% of the variance in health behaviours, indicating that unplanned, socially reactive decisions are important to account for. Prototype favourability and similarity explained 1.1% of additional variance in behaviour over and above reasoned intentions, while willingness explained 1.4%. Van Lettow, de Vries, Burdorf, and van Empelen (2016) examined the weighted correlations between prototype perceptions and health behaviours. Overall, prototype favourability (*r* = 0.20) and similarity (*r* = 0.27) were associated with behaviour, with small effect sizes (Cohen, 1992). Prototype similarity (*r* = 0.34) was more strongly related than prototype favourability (*r* = 0.15) to health protective behaviour. These findings suggest social‐reactive constructs could have a small but important explanatory role in physical activity behaviour.

No population studies have examined associations between adolescents’ active and inactive prototype perceptions and physical activity, and evidence from a few small studies is inconclusive. Broad ‘exerciser’ and ‘non‐exerciser’ prototype perceptions were associated with *intentions* to exercise (Rivis, Sheeran, & Armitage, 2006), but ‘cycling’ prototype perceptions did not explain variance in *intentions* to cycle to school over and above TPB variables (Frater, Kuijer, & Kingham, 2017). Prototype perceptions did explain additional variance in *objectively measured activity* over TPB variables (Wheatley, Johansen‐Berg, Dawes, & Davies, 2020).

### Aims and hypotheses

We explored the utility of a dual‐process approach for explaining physical activity and change in physical activity behaviour. First, we examined cross‐sectional relationships andaimed to measure the relative strength of associations between physical activity and constructs in the TPB and the PWM, and the variance explained by the PWM over and above TPB variables. Specifically, we hypothesized that active and inactive prototype perceptions and willingness would have significant associations with physical activity, and that they would explain additional variance in behaviour over and above TPB constructs. We also investigated physical activity behaviour‐change during adolescence by examining longitudinal relationships: here, we aimed to measure the relative strength of associations between change in physical activity and change in TPB and PWM constructs, and the variance explained by the change in PWM variables over and above change in TPB variables. We predicted a 1‐year change (decline) in physical activity would be associated with 1‐year changes in prototype perceptions and willingness, and that these change measures would explain additional variance over and above changes in TPB measures. Finally, we explored whether these relationships varied with sex and SES.

## Method

### Design

A cross‐sectional and longitudinal analysis of baseline (June–September 2017) and follow‐up (May–September 2018) data from the Fit to Study cluster‐randomized controlled trial, registered with ClinicalTrials.gov, NCT03286725. Participants in the intervention arm were asked to complete 20 min of vigorous physical activity per hour of physical education (PE) in school, while the control arm did PE as usual. In this study, all participants were treated as one cohort, but we controlled for school clustering and trial treatment status (intervention or control) in the analyses. Trial recruitment, consent procedures, and methodology are reported elsewhere (Wassenaar et al., 2019) in line with the Consolidated Standards for Reporting Trials. The trial was approved by the Central University Research Ethics Committee of University of Oxford (Registration No. R48879/RE001) and all research was performed in accordance with the Declaration of Helsinki.

### Participants

Participants were English Year 7 pupils (aged 11–13) at baseline. Schools provided participants’ sex, birth date, and their eligibility for free school meals (FSM), an indicator of socioeconomic disadvantage (Taylor, 2017).

At baseline, 9,699 participants (female = 5,836; 60.1%; eFSM = 1,391; 14.3%; trial intervention condition = 4,734; 48.8%) from 82 schools completed the questionnaire; 95.2% did so before the summer vacation and 93.3% were assessed in school. At follow‐up, the longitudinal sample was 4,632 participants from 52 schools (female = 3,049; 65.8%; eFSM = 584; 12.6%; trial intervention condition = 1,899; 40.1%; 20 schools) had completed assessments at both time‐points; 98.1% of follow‐up assessments took place before the vacation and 83.0% were in school. Mean age at the start of Year 8 was 12.5(*SD* 0.29) years in both samples.

The full sample available at the Fit to Study trial’s baseline was school *n* = 93; pupil *n* = 16,017; male = 7,056 (44.0%); and FSM = 3,068 (19.1%). Loss to follow‐up and data cleaning methods are reported in Figure S1.

### Outcome measures

Participants completed questionnaires on school computers during class time, or otherwise at home. We used short or single‐item measures where possible to minimize participant burden. All constructs were measured on a seven‐point scale where higher values indicate a more positive response, allowing comparisons of effect sizes within (but not between) models. We used the same action, context, and school‐term time‐frame – ‘for an hour every day during a typical week in term’ (represented by ellipses in the descriptions below) – to emphasize thoughts and beliefs about travel to school, PE lessons, and after‐school sport. We calculated Cronbach’s α as a measure of internal consistency for variables that were the mean score of three or more items and Spearman’s *r* for variables that were the mean of two items.

#### Physical activity variables

Physical activity (past week) was measured with a validated, single‐item measure (Scott, Morgan, Plotnikoff, & Lubans, 2015): ‘In the past week, on how many days have you done a total of 60 min or more of physical activity, which was enough to raise your breathing rate? This may include sport, exercise, and brisk walking or cycling for fun, or to get to and from places’ (0–7 days).

Past behaviour (habitual physical activity) was measured with a single self‐report item (Hagger, Chatzisarantis, Biddle, & Orbell, 2001): ‘Thinking about the past 6 months, how often have you been physically active for an hour every day during a typical week in term?’ (1 = ‘never’ to 7 = ‘always’).

#### TPB variables

These were developed in line with established theoretical principles (Francis et al., 2004). The intention was measured with a single item: ‘*I intend to be physically active…*’ (1 = definitely no’, 7 = ‘definitely yes’). The attitude was the mean score of three items (α = 0.82) on a visual analogue scale. The stem was ‘For me, being physically active…would be:’ and the response options were boring‐fun; stressful‐relaxing; and dissatisfying‐satisfying. In line with previous PWM studies investigating the impact of reactive over planned constructs (Todd et al., 2016), we measured subjective norms. These were the mean score of four items, α = 0.71), capturing family and friends’ injunctive norms with ‘My family/most of my friends think I should be physically‐active…’ and descriptive norms with ‘My family/most of my friends will be physically active…’ (1= ‘strongly disagree’, 7 = ‘strongly agree’). PBC was measured with two items, capturing self‐efficacy: ‘If I wanted to, I am confident that I could be physically‐active …’ and control: ‘Whether or not I am physically‐active… is entirely up to me’ (1 = ‘strongly disagree’, 7 = ‘strongly agree’). The correlation between these items was low (Spearman’s *r* = 0.25) so they were included as separate measures (capacity and autonomy) in analyses.

#### PWM variables

Perceptions of active and inactive prototypes were measured in line with previous research (Gibbons, Gerrard, & McCoy, 1995). Favourability was rated against social characteristics or attributes that adolescents understand, share and apply to active and inactive prototypes. These characteristics were four, syllable‐matched adjectives (*confident, popular, determined, and attractive*) drawn from qualitative research in this age group (Wheatley, Davies, & Dawes, 2017). Participants were asked to consider active (and, separately, and inactive) prototypes: ‘Think of someone your age who is physically active (inactive) …’ and indicate ‘how far the following words describe your image of this person?’ (1 = ‘not at all’ to 7 = ‘extremely’). Prototype favourability was the mean score across the four attributes (active/favourability α = 0.71; inactive/favourability α = 0.80). The similarity was measured with a single item: ‘In general, how similar are you to the type of person who is physically active (inactive)…?’ (1 = ‘not at all’ to 7 = ‘extremely’).

Willingness was measured by presenting participants with scenarios in which they could choose to be active in a social setting, based on previous work (Gerrard et al., 2002). The first scenario was, ‘It's lunchtime at school and the teachers have organised an obstacle race on the playing field for a challenge. Some students are taking part and others are watching’. Participants were asked ‘How willing are you to be physically active by doing the obstacle race?’ and then (score reversed) ‘How willing are you to avoid being physically active by watching the obstacle race?’ (1 = ‘very unwilling’ to 7 = ‘very willing’). The second scenario, followed by the same two questions, was ‘It’s nearly the end of the school year. To celebrate, the teachers have organised some activities. You have been offered the option to either spend the afternoon doing some sort of physical activity such as canoeing or ice‐skating – or going home early.’ Willingness was the mean score on four items (α = 0.79).

### Statistical analyses

Cross‐sectional analyses included participants completing all baseline measures, and longitudinal analyses included those who completed measures at baseline and 1‐year follow‐up.

#### Baseline descriptive statistics

To examine relationships and check for multi‐collinearity (indicated where *r > *0.7; Cohen, 1988), we calculated Spearman’s *r* correlations between all model variables.

#### Cross‐sectional analyses of baseline data

Our aims were to compare the strength of associations between TPB and PWM constructs and physical activity behaviour, and to measure the variance explained by PWM constructs over and above TPB variables. We developed a stepwise multilevel linear regression model, with random intercepts to account for school clusters. We entered past (habitual) behaviour, sex, and FSM at step 1 because they are non‐modifiable, followed by TPB variables at step two, and prototype perceptions and willingness at step 3: this hierarchy is in line with previous analyses (Todd, Kothe, Mullan, & Monds, 2014). In steps 2 and 3, we included three‐way and also two‐way interaction terms of sex and FSM with each of the model variables to explore whether the strength of associations varied with sex and SES: where interactions were non‐significant they were removed and the model was re‐run. Each step of the model controlled for pupil age, time of assessment (summer term, school holiday, or autumn term), and place of assessment (school or home). We bootstrapped 95% confidence intervals to account for the non‐normal distribution of model residuals. We calculated the intra‐class correlation coefficient (ICC) at the final step to examine the variance in physical activity explained by school clusters. The Goodness‐of‐fit of successive models was estimated with a likelihood ratio test and multicollinearity was further checked by calculating the variance inflation factor (VIF) of each variable in the final models, taking VIF > 5 to indicate a problem.

#### Longitudinal analyses of follow‐up data

To examine change in variables over time, we estimated the main effect of time on physical activity and all model variables in the longitudinal sample using a repeated‐measures design in a multilevel setting, accounting for school clusters and trial treatment status (intervention or control).

In the longitudinal sample, changes in all variables were calculated by subtracting follow‐up scores from baseline scores. As with cross‐sectional analyses, we aimed to compare the strength of associations between changes in measured TPB and PWM constructs and change physical activity, and to measure the variance explained by PWM constructs over and above TPB variables. As with cross‐sectional analyses, we developed a three‐step multilevel linear model with random intercepts for school clusters, controlled for treatment status (intervention or control), age, and also time and place of assessments at both baseline and follow‐up. Non‐significant interaction terms of sex and FSM were excluded as before. Confidence intervals were bootstrapped, goodness‐of‐fit, VIF, and ICC were calculated as before.

Given the large sample size, we took *p* < .005 to indicate statistical significance in all models, in line with calls for more stringent alpha levels (Johnson, 2013); where there were significant interactions we explored these by plotting the data. Analyses were conducted in R(3.5.1) with the lme4, glmm, ggplot2, and boot packages.

#### Sensitivity analyses

To explore any potential bias related to trial participation, we took participants assigned to the control arm of the trial and re‐ran cross‐sectional analyses (*n* = 4,965) and longitudinal analyses (*n* = 2,773) in these groups.

## Results

### Baseline descriptive statistics

In the baseline sample, all variables were significantly correlated (*p* < .005; see Table 1) except inactive prototype favourability with attitude (*p = *.02), with subjective norms (*p* = .54) and with active prototype similarity (*p = *.13); and between inactive prototype similarity and PBC (autonomy; *p* = .41). Physical activity was strongly correlated with past behaviour and intention (Cohen, 1988), but there was no evidence of multi‐collinearity.

**Table 1:** Baseline Spearman’s *r* correlation of TPB and PWM variables (*n* = 9,699)

Variables	1	2	3	4	5	6	7	8	9	10	11	12
1. Past behaviour	1											
2. Intention	0.65	1										
3. Attitude	0.55	0.62	1									
4. Subjective norms	0.42	0.49	0.43	1								
5. PBC: capacity	0.52	0.59	0.56	0.43	1							
6. PBC: autonomy	0.15	0.14	0.16	0.13	0.22	1						
7. Favourable/active	0.23	0.27	0.28	0.28	0.27	0.15	1					
8. Similar/active	0.57	0.55	0.51	0.37	0.46	0.13	0.27	1				
9. Favourable/inactive	−0.05	−0.05	−0.02	−0.01	−0.06	0.03	0.06	0.02	1			
10. Similar/inactive	−0.33	−0.31	−0.30	−0.17	−0.29	−0.01	−0.09	−0.29	0.39	1		
11. Willingness	0.45	0.48	0.52	0.30	0.48	0.11	0.23	0.45	−0.11	−0.36	1	
12. Physical activity	0.61	0.54	0.47	0.37	0.44	0.12	0.21	0.48	−0.05	−0.30	0.40	1

Note

All correlations *p* < .005 except attitude and inactive/favourable *p* = .02; subjective norms and inactive/favourable *p* = .539; active/similar and inactive/favourable *p* = .125 and PBC2/inactive/similar *p* = .414.

### Cross‐sectional analyses of baseline data

In the full model, there were significant associations in the expected direction between physical activity and all variables except PBC (autonomy) and prototype favourability (see Table 2). Physical activity had the strongest association with past behaviour (β = 0.48, 95% CI 0.45 to 0.51, *p* < .001), followed by intention (β = 0.15, 95% CI 0.12 to 0.18, *p* < .001) and similarity to active prototypes (β = 0.13, 95% CI 0.10 to 0.15, *p* < .001).

**Table 2:** Estimates of cross‐sectional associations between individual‐level variables and baseline physical activity (*n* = 9,669) and longitudinal (*n* = 4,632)

Predictor variables	Baseline: cross‐sectional analysis				Follow‐up: longitudinal analysis			
	*R* ^2^	β	2.5% CI	97.5% CI	*R* ^2^	β	2.5% CI	97.5% CI
*Step 1*	0.377				0.135			
Habitual activity		0.82**	0.80	0.84		0.52**	0.48	0.56
Sex^a^		−0.26**	−0.33	−0.20		−0.03	−0.15	0.09
eFSM^b^		−0.05	−0.14	0.04		0.03	−0.13	0.20
*Step 2*	0.426				0.179			
Habitual activity		0.54**	0.51	0.57		0.35**	0.31	0.40
Sex^a^		−0.20**	−0.27	−0.14		−0.02	−0.14	0.09
eFSM^b^		0.03	−0.05	0.12		0.03	−0.13	0.19
Intention		0.19**	0.16	0.21		0.17**	0.13	0.21
Attitude		0.14**	0.11	0.17		0.16**	0.11	0.21
Subjective norms		0.09**	0.06	0.11		0.04	−0.01	0.08
PBC: capacity		0.09**	0.07	0.12		0.08**	0.04	0.12
PBC: autonomy		0.00	−0.02	0.02		0.01	−0.03	0.04
*Step 3*	0.438				0.189			
Habitual activity		0.48**	0.45	0.51		0.32**	0.28	0.36
Sex^a^		−0.20**	−0.26	−0.14		−0.02	−0.13	0.10
eFSM^b^		0.04	−0.04	0.13		0.04	−0.12	0.20
Intention		0.15**	0.12	0.18		0.15**	0.11	0.19
Attitude		0.08**	0.05	0.11		0.13**	0.08	0.18
Subjective norms		0.08**	0.05	0.10		0.03	−0.02	0.07
PBC: capacity		0.06**	0.03	0.09		0.06*	0.02	0.10
PBC: autonomy		0.00	−0.02	0.02		0.01	−0.02	0.04
Active favourable		0.03	−0.00	0.05		0.02	−0.02	0.06
Active similar		0.13**	0.10	0.15		0.10**	0.06	0.13
Inactive favourable		0.00	−0.02	0.03		−0.01	−0.04	0.03
Inactive similar		−0.06**	−0.08	−0.04		−0.05**	−0.08	−0.02
Willingness		0.07**	0.04	0.10		0.05	0.01	0.10

Note

Fully adjusted multilevel model including covariates of age, sex, eFSM, term/place of measurement, and school effects, with confidence intervals bootstrapped.

^a^ Reference category: male.

^b^ Reference category: not eligible for FSM.

* *p* < .005; **p < .001.

At step 1 of the model, past (habitual) behaviour, sex, and FSM explained 37.7% of the variance (see Table 2). There were no interactions between either sex or FSM and any of the predictor variables in either of the subsequent model steps, so these terms were excluded from the final models. Adding intention, attitude, subjective norms, and PBC measures at step 2 explained a small amount of additional variance, 4.9%, and improved the model fit, *Χ* ^2^ (11,16) = 783, *p* < .001. Adding prototype perceptions and willingness at step 3 also explained a small amount of additional variance, 1.2%, and further improved the model fit, *Χ* ^2^ (16,21) = 218, *p* < .001, at step 3, ICC = 0.04. VIF < 2.4 for all variables.

### Longitudinal analyses of follow‐up data

In the longitudinal sample, there was a significant main effect of time on physical activity, β = −0.16, *p* < .001: mean active days per week were lower at follow‐up (4.3) than at baseline (4.5). Scores on all individual‐level variables apart from PBC (autonomy) were significantly lower (less positive) at follow‐up (Table 3).

**Table 3:** Comparison of scores at baseline and follow‐up using multilevel regression (*n* = 4,632)

Variables	Baseline	Follow‐up	Effect of time‐point
	Mean (*SD*)	Mean (*SD*)	β^a^
Past (habitual) behaviour	5.1 (1.4)	5.0 (1.4)	−0.13*
Intention	5.4 (1.5)	5.1 (1.7)	−0.25*
Attitude	5.0 (1.2)	4.9 (1.2)	−0.12*
Subjective norms	4.8 (1.1)	4.7 (1.2)	−0.13*
PBC: capacity	5.8 (1.4)	5.7 (1.4)	−0.11*
PBC: autonomy	5.4 (1.4)	5.5 (1.4)	0.05
Favourable/active	5.2 (1.0)	5.1 (1.0)	−0.06*
Similar/active	4.7 (1.5)	4.5 (1.5)	−0.21*
Favourable/inactive	3.6 (1.3)	3.4 (1.3)	−0.24*
Similar/inactive	3.0 (1.7)	3.2 (1.7)	0.17*
Willingness	5.1 (1.3)	4.8 (1.4)	−0.34*
Physical activity	4.5 (1.9)	4.3 (1.9)	−0.16*

^a^ Repeated‐measures comparison in the follow‐up sample only; analysis is a multilevel regression measuring the effect of time‐point (baseline or follow‐up) on each variable, controlling for treatment status and school random effects.

* *p < *.005.

In the full model, there were no significant interactions between either sex or FSM and any of the predictor variables at the *p* < .005 level, although the interaction between sex and active prototype similarity approached significance (β = −0.11, 95% CI −0.19 to −0.03, *p* = .008): the positive relationship between change in prototype perception and change in physical activity was stronger for boys than girls. When the model was re‐run without any interaction terms, there were significant associations in the expected direction between change in physical activity and change in past (habitual) behaviour, intention, attitude, PBC (capacity), and prototype similarity only (see Table 2). Change in past (habitual) behaviour had the strongest association with change in physical activity (β = 0.32, 95% CI 0.28 to 0.36, *p* < .001), followed by change in intention (β = 0.15, 95% CI 0.11 to 0.19, *p* < .001), change in attitudes (β = 0.13, 95% CI 0.08 to 0.18, *p* < .001) and change in similarity to active prototypes (β = 0.10, 95% CI 0.06 to 0.13, *p* < .001).

At step 1 of the model, change in past (habitual) activity, sex, and FSM explained 13.5% of variance in physical activity behaviour change (see Table 2). Adding change in intention, attitude, subjective norms and PBC measures at step 2 explained an additional 4.4% of variance, and improved the model fit, *X* ^2^ (6,11) = 241, *p* < .001. Adding prototype perceptions and willingness at step 3 explained a small amount of additional variance, 1.0%, and further improved the model fit, *X* ^2^ (11,16) = 62.5, *p* < .001. At step 3, ICC = 0.01. VIF < 2 for all variables.

### Sensitivity analyses

In the cross‐sectional analyses, the strength of association between physical activity and model constructs, and the additional variance explained by PWM variables, were very similar in the control‐only sample (see Table S1). In the longitudinal analyses, the strength of associations between change in model constructs and physical activity behaviour change were broadly similar in the control‐only sample: beta values for habitual activity, intention, attitude, and active prototype similarity were slightly lower, while PBC (capacity) was slightly higher than in the full sample. Although the total variance explained by the final model was slightly greater in the full sample, the additional variance in physical activity explained by PWM variables was almost the same.

## Discussion

This study showed that a dual‐process health behaviour model incorporating social‐reactive constructs could be more useful for explaining and predicting young adolescents’ physical activity in a population sample than models based on rational expectations alone. Although the strength of associations and the additional variance explained by PWM constructs were relatively small, they are comparable with studies of other health behaviours (Todd et al., 2016), suggesting the model may be a useful framework for physical activity interventions. In the cross‐sectional analyses, days per week of activity were associated with prototype similarity and willingness. PWM constructs explained 1.2% of additional variance over and above TPB variables and established correlates of physical activity, including past behaviour and sex, whereas Todd et al. (2016) found that across a range of health behaviours, favourability, similarity, and willingness accounted for 2.5% of additional variance over and above intention only. In the analyses of follow‐up data, physical activity declined over the year, and this change was linked with a decline in similarity to active prototypes and an increase in similarity to inactive prototypes. Changes in PWM measures explained unique variance in activity behaviour change.

### Cross‐sectional analyses of baseline data

Our findings suggest prototype similarity is an important correlate of adolescent physical activity. At baseline, regression analysis showed that active prototype similarity was more strongly related to activity than attitudes, norms, and PBC measures from the TPB, which are all considered useful for modelling behaviour (Hagger et al., 2002; McEachan et al., 2011). Furthermore, this relationship existed not only after controlling for sex and SES – established as correlates of physical activity (Sterdt et al., 2014) – but also while accounting for past (habitual) behaviour in the model. Inactive prototype similarity was also linked with physical activity, but the strength of this negative relationship was weaker. For comparison, and considering the behaviour most frequently explored with the PWM, Todd et al. (2016) found that the strength of the relationship between drinker prototype perceptions (favourability and similarity) and alcohol consumption across all studies was β = 0.145, suggesting that active prototype similarity this is a useful construct for predicting adolescent activity behaviour.

These are striking results because it counters the criticism that measures of prototype similarity and habitual behaviour are tapping into something very similar in the sense that individuals self‐identify as active (or inactive) only because they have been active (or inactive) in the past (Ajzen, 2011; Gibbons & Gerrard, 2016). These findings, which align with evidence that actor prototypes are typically stronger predictors of health intentions than abstainer prototypes (Rivis et al., 2006), could be because an active self‐identity – a positive image including attractiveness and confidence, for example – is more salient than an image involving the absence of these qualities.

Notably, there was no significant relationship between physical activity and prototype favourability, a measure shown to be more closely aligned with potentially appealing health‐risk behaviours such as moderate alcohol consumption than with health‐promoting behaviours such as nutritious food choices (Todd et al., 2016; Todd & van Lettow, 2016). This is also an important finding because it suggests that, in this age group, recognizing the positive characteristics of the type of person who is active (and the less positive characteristics of inactive types) does not appear to significantly influence behaviour.

Physical activity was also associated with willingness to be active in social situations, although this relationship was weaker than its link with reasoned intention. Nevertheless, this is an important finding because behavioural willingness is typically associated with health‐risk behaviours such as smoking and drinking (Todd & van Lettow, 2016), in which young people may not plan to participate, but become open to doing so in reaction to social circumstances, as they weigh up competing for social and health risks. It has been suggested that it can be socially risky for an adolescent to *not* engage in a risky behaviour such as drinking at a party (Gibbons & Gerrard, 2016), but the influence of willingness here indicates there could also be a social risk attached to engaging in physical activity, a health behaviour. Active adolescents can, in certain circumstances, be perceived negatively (Wheatley et al., 2017). Further research is needed to examine the extent to which *unwillingness* to be active – in reaction to a social situation in which exercise might be ‘uncool’ or ‘showing off’, for example – is an influential factor.

### Longitudinal analyses of follow‐up data

Mean days per week of physical activity declined over the year, and scores on all TPB and PWM variables apart from PBC (autonomy) were also significantly less positive at follow‐up, highlighting the temporal instability of these individual differences during adolescence. This is the first study to examine longitudinal associations in this framework as they relate to physical activity. Change in *past behaviour*, in itself a non‐modifiable construct and therefore less important when considering intervention targets, is the strongest predictor of change in physical activity. Yet it is striking that change in similarity to an active image has a stronger relationship with change in behaviour than do changing norms or PBC measures during this period of rapid psychosocial development (Blakemore & Mills, 2014), and that similarity to inactive images was also related to change in days per week of activity, although this relationship was weaker than other associations between model constructs and physical activity.

One explanation for the relationship between change in similarity to active prototypes and change in activity over time is that adolescents’ self‐images are becoming more developed and therefore more influential: self‐consistency becomes a powerful motivator of health behaviour through adolescence (Aloise‐Young, Hennigan, & Graham, 1996). Willingness – the construct with the largest main effect of time – was not related to change in physical activity over a year, which is surprising given the strength of its association with activity in the cross‐sectional analyses. One explanation might be that ‘willingness’ in the scenarios presented is capturing something other than perceptions of social risks, such as changing affective attitudes or autonomy. Further, there is evidence for a developmental shift from reactive to planned behaviour from early to middle adolescence (Pomery, Gibbons, Reis‐Bergan, & Gerrard, 2009), suggesting that intentions could become more influential than willingness over time.

### Practical implications for interventions

Our overall aim was to explore the extent to which a dual‐process framework that accounts for impulsive reactions to social situations could help inform adolescent physical activity interventions. The strength of the relationship between prototype similarity and physical activity – and evidence that the construct can change over time – indicates that self‐identity in the context of physical activity could be a useful target for intervention. But highlighting aspirational social qualities of physically active people – such as attractiveness or determination – may be a less fruitful approach, given the weak relationship between behaviour and prototype favourability.

One type of intervention that targets the importance of image similarity uses the concept of the ‘possible self’, which refers to self‐knowledge about potential life outcomes given individual traits and behaviours: the accompanying hopes and fears for the future are said to motivate behavioural regulation (Markus & Nurius, 1986). ‘Possible selves’ have been used to explore and manipulate physical activity with some success (Murru & Ginis, 2010; Ouellette et al., 2005; Perras, Strachan, & Fortier, 2016). In this paradigm, participants are encouraged to reflect on the costs and benefits of long‐term activity (or inactivity): for example, one intervention increased physical activity among adults by asking participants to think about their future selves as a regular exercisers, to consider what images come to mind, and to imagine the barriers and facilitators to achieving this possible self (Strachan, Marcotte, Giller, Brunet, & Schellenberg, 2017). Adolescence is a period during which future orientation, motivation, and planning skills are developing (Nurmi, 1991) so this approach may have potential among younger pupils too

Other approaches recognize that individuals identify not only as themselves but also as members of a group (Tajfel, 1982). Some applied physical activity studies have shown that individuals are more likely to engage in health behaviours to the extent they match the content of a salient social group’s identity (Kwasnicka et al., 2020). One social group with which adolescents identify is their school (Reynolds, Lee, Turner, Bromhead, & Subasic, 2017), so this approach could be extended with a whole‐school intervention promoting a more inclusive active image that incorporates active commuting and active lesson breaks, for example. The prototype‐behaviour relationship did not vary significantly with either sex or SES, so such interventions might be suitable for adolescents from a range of backgrounds.

### Strengths and limitations

A key strength of this study is the large longitudinal data set, which allowed us to explore the extent to which a large number of psychosocial constructs and covariates of sex and SES were related to physical activity in a broad sample of adolescents. Although our study cannot support conclusions about causality, its longitudinal design strengthens the case that targeting variables in the dual‐process PWM could lead to behaviour change.

A key limitation is that the proportion of participants lost to follow‐up introduced bias. Imputing missing data for multilevel modelling using basic techniques risks introducing further bias: we judged that accepting missingness as a limitation, and running a sensitivity analysis, represented the most transparent interpretation of the data. Although we adjusted regression models for a range of confounding factors, we acknowledge more may be present.

Constructs in the TPB and PWM have indirect effects on behaviour (see Figure 1), but we chose not to explore these. Numerous studies have also demonstrated direct links between model constructs and behaviour (Armitage & Conner, 2001; Cooke, Dahdah, Norman, & French, 2016; Todd et al., 2016), and we chose to focus on these because the purpose of the study was to find modifiable constructs for interventions to increase physical activity. Further studies would develop this work by exploring indirect effects.

We used single‐item self‐report measures of physical activity, intention, and prototype similarity to minimize participant burden during Fit to Study. Two items measuring PBC showed low internal consistency at baseline and follow‐up, suggesting physical activity capacity and autonomy are distinct and unrelated concepts in adolescent samples. Willingness measures showed good internal consistency at both time points, but it is possible that self‐report measures, requiring conscious deliberation, may not accurately capture the impulsive dimension of behavioural willingness (Davies, Paltoglou, & Foxcroft, 2017; Fishbein, 2008).

### Conclusions

This large‐sample study adds to evidence that a dual‐process health behaviour model could be more useful for explaining and predicting young adolescents’ physical activity than models based on rational expectations alone. The strength of the association between physical activity behaviour and active prototype similarity in both cross‐sectional and longitudinal analyses suggests that self‐image and social identity in the context of physical activity could be useful targets for intervention in this age group. Behaviour‐change interventions promoting an active self‐image could be tested for effects on physical activity among young adolescents.

## Conflicts of interest

All authors declare no conflict of interest.

## Author contribution

**Catherine M. Wheatley:** Formal analysis (equal); Investigation (equal); Methodology (equal); Writing – original draft (equal). **Thomas Wassenaar:** Data curation (equal); Formal analysis (equal); Investigation (equal); Writing – review & editing (equal). **Nick Beale:** Data curation (equal); Project administration (equal); Writing – review & editing (equal). **Piergiorgio Salvan:** Formal analysis (equal); Investigation (equal); Writing – review & editing (equal). **Helen Dawes:** Conceptualization (equal); Funding acquisition (equal); Writing – review & editing (equal). **Emma Davies:** Investigation (equal); Methodology (equal); Writing – review & editing (equal). **Heidi Johansen‐Berg:** Conceptualization (equal); Formal analysis (equal); Funding acquisition (equal); Writing – review & editing (equal).

## Supporting information


**Figure S1**. Flow chart showing Fit to Study sample at baseline and follow‐up.

**Table S1**. Associations between model variables and physical activity, and variance explained, in control sample only at baseline (*n* = 4,965) and follow‐up (*n* = 2,773).

**Table S2**. Provide the complete and correct author list, in order, as it should be appear on the article (attach and additional sheet if more space is needed).

**Table S4**. Provide a list of the individuals and/or representatives of multi‐author collaborative or consortia group to be REMOVED from the author list as it was originally provided for the article cited in Table 1.

## Data Availability

The full Fit to Study trial data are archived and available from the corresponding author on reasonable request. The datasets generated and here analysed are not publicly available due to the sensitivity of the data.

## References

[bjhp12582-bib-0001] Ajzen, I. (1985). From intentions to actions: A theory of planned behavior. In J. Kuhl & J. Beckmann (Eds.), Action control. SSSP springer series in social psychology. Berlin: Springer.

[bjhp12582-bib-0002] Ajzen, I. (2011). The theory of planned behaviour: Reactions and reflections. Health Psychology Review, 26(9), 1113–1127. 10.1080/08870446.2011.613995 21929476

[bjhp12582-bib-0003] Ajzen, I. , & Schmidt, P. (2020). The handbook of behavior change. In M. Hagger , L. Cameron , K. Hamilton & N. Hankonen (Eds.), Cambridge handbooks in psychology. Cambridge, UK: Cambridge University Press.

[bjhp12582-bib-0004] Aloise‐Young, P. A. , Hennigan, K. M. , & Graham, J. W. (1996). Role of the self‐image and smoker stereotype in smoking onset during early adolescence: A longitudinal study. Health Psychology, 15, 494–497. 10.1037/0278-6133.15.6.494 8973931

[bjhp12582-bib-0005] Armitage, C. J. , & Conner, M. (2001). Efficacy of the theory of planned behaviour: A meta‐analytic review. British Journal of Social Psychology, 40, 471–499. 10.1348/014466601164939 11795063

[bjhp12582-bib-0006] Blakemore, S.‐J. , & Mills, K. L. (2014). Is adolescence a sensitive period for sociocultural processing? Annual Review of Psychology, 65, 187–207. 10.1146/annurev-psych-010213-115202 24016274

[bjhp12582-bib-0068] Buchan, D. S. , Ollis, S. , Thomas, N. E. , & Baker, J. S. (2012). Physical activity behaviour: An overview of current and emergent theoretical practices. Journal of Obesity, 2012(1‐11). 10.1155/2012/546459 PMC3388376 22778918

[bjhp12582-bib-0007] Cohen, J. (1988). Statistical power analysis for the behavioral sciences (2nd ed.). New York, NY: Routledge.

[bjhp12582-bib-0008] Cohen, J. (1992). A power primer. Psychological Bulletin, 112(1), 155–159. 10.1037/0033-2909.112.1.155 19565683

[bjhp12582-bib-0009] Conner, M. , & Sparks, P. (2005). Theory of planned behaviour and health behaviour. In M. Conner & P. Norman (Eds.), Predicting health behavior: Research and practice with social cognition models. New York, NY: Open University Press, McGraw‐Hill Education.

[bjhp12582-bib-0010] Cooke, R. , Dahdah, M. , Norman, P. , & French, D. P. (2016). How well does the theory of planned behaviour predict alcohol consumption? A systematic review and meta‐analysis. Health Psychology Review, 10, 148–167. 10.1080/17437199.2014.947547 25089611 PMC4867851

[bjhp12582-bib-0011] Dahl, R. E. , Allen, N. B. , Wilbrecht, L. , & Suleiman, A. B. (2018). Importance of investing in adolescence from a developmental science perspective. Nature, 554, 441–450. 10.1038/nature25770 29469094

[bjhp12582-bib-0012] Davies, E. L. , Paltoglou, A. E. , & Foxcroft, D. R. (2017). Implicit alcohol attitudes predict drinking behaviour over and above intentions and willingness in young adults but willingness is more important in adolescents: Implications for the Prototype Willingness Model. The British Journal of Health Psychology, 22, 238–253. 10.1111/bjhp.12225 27925361

[bjhp12582-bib-0013] Elliott, M. A. , McCartan, R. , Brewster, S. E. , Coyle, D. , Emerson, L. , & Gibson, K. (2017). An application of the prototype willingness model to drivers’ speeding behaviour. European Journal of Social Psychology, 47, 735–747. 10.1002/ejsp.2268

[bjhp12582-bib-0014] Farooq, A. , Martin, A. , Janssen, X. , Wilson, M. G. , Gibson, A. , Hughes, A. , & Reilly, J. J. (2020). Longitudinal changes in moderate‐to‐vigorous‐intensity physical activity in children and adolescents: A systematic review and meta‐analysis. Obesity Reviews, 21(1), e12953. 10.1111/obr.12953 31646739 PMC6916562

[bjhp12582-bib-0015] Fishbein, M. (2008). A reasoned action approach to health promotion. Medical Decision Making, 28, 834–844. 10.1177/0272989X08326092 19015289 PMC2603050

[bjhp12582-bib-0016] Francis, J. , Eccles, M. P. , Johnston, M. , Walker, A. E. , Grimshaw, J. M. , Foy, R. , … Bonetti, D. (2004). Constructing questionnaires based on the theory of planned behaviour: A manual for health services researchers. Centre for Health Services Research, University of Newcastle upon Tyne.

[bjhp12582-bib-0017] Frater, J. , Kuijer, R. , & Kingham, S. (2017). Why adolescents don’t bicycle to school: Does the prototype/willingness model augment the theory of planned behaviour to explain intentions? Transportation Research Part F: Traffic Psychology and Behaviour, 46, 250–259. 10.1016/j.trf.2017.03.005

[bjhp12582-bib-0018] Gardner, B. , de Bruijn, G.‐J. , & Lally, P. (2011). A systematic review and meta‐analysis of applications of the self‐report habit index to nutrition and physical activity behaviours. Annals of Behavioral Medicine, 42, 174–187. 10.1007/s12160-011-9282-0 21626256

[bjhp12582-bib-0019] Gerrard, M. , Gibbons, F. X. , Houlihan, A. E. , Stock, M. L. , & Pomery, E. A. (2008). A dual‐process approach to health risk decision making: The prototype willingness model. Developmental Review, 28(1), 29–61. 10.1016/j.dr.2007.10.001

[bjhp12582-bib-0020] Gerrard, M. , Gibbons, F. X. , Reis‐Bergan, M. , Trudeau, L. , Vande Lune, L. S. , & Buunk, B. (2002). Inhibitory effects of drinker and nondrinker prototypes on adolescent alcohol consumption. Health Psychology, 21, 601–609. 10.1037/0278-6133.21.6.601 12433013

[bjhp12582-bib-0021] Gibbons, F. X. , & Gerrard, M. (1995). Predicting young adults’ health risk behavior. Journal of Personality and Social Psychology, 69, 505–517. 10.1037/0022-3514.69.3.505 7562392

[bjhp12582-bib-0022] Gibbons, F. X. , & Gerrard, M. (2016). Reactions to the meta‐analyses of the Prototype Willingness Model. Health Psychology Review, 10(1), 44–46. 10.1080/17437199.2015.1116020 26541896

[bjhp12582-bib-0023] Gibbons, F. X. , Gerrard, M. , & Lane, D. J. (2003). A social reaction model of adolescent health risk. In J. Suls & K. A. Wallson (Eds.), Social psychological foundations of health and illness (pp. 107–136). Oxford: Blackwell Publishing.

[bjhp12582-bib-0024] Gibbons, F. X. , Gerrard, M. , & McCoy, S. B. (1995). Prototype perception predicts (lack of) pregnancy prevention. Personality and Social Psychology Bulletin, 21(1), 85–93. 10.1177/0146167295211009

[bjhp12582-bib-0025] Gibbons, F. X. , Houlihan, A. E. , & Gerrard, M. (2009). Reason and reaction: The utility of a dual‐focus, dual‐processing perspective on promotion and prevention of adolescent health risk behaviour. British Journal of Health Psychology, 14, 231–248. 10.1348/135910708X376640 19026095

[bjhp12582-bib-0026] Gibbons, F. X. , Kingsbury, J. H. , Gerrard, M. , & Wills, T. A. (2011). Two ways of thinking about dual processing: A response to Hofmann, Friese and Wiers (2008). Health Psychology Review, 5, 158–161. 10.1080/17437199.2010.541823

[bjhp12582-bib-0027] Guthold, R. , Stevens, G. A. , Riley, L. M. , & Bull, F. C. (2020). Global trends in insufficient physical activity among adolescents: A pooled analysis of 298 population‐based surveys with 1 6 million participants. The Lancet Child & Adolescent Health, 4(1), 23–35. 10.1016/S2352-4642(19)30323-2 31761562 PMC6919336

[bjhp12582-bib-0028] Hagger, M. S. , Chatzisarantis, N. L. D. , & Biddle, S. J. H. (2002). A meta‐analytic review of the theories of reasoned action and planned behavior in physical activity: Predictive validity and the contribution of additional variables. Journal of Sport and Exercise Psychology, 24(1), 3–32. 10.1123/jsep.24.1.3

[bjhp12582-bib-0029] Hagger, M. S. , Chatzisarantis, N. , Biddle, S. J. H. , & Orbell, S. (2001). Antecedents of children’s physical activity intentions and behaviour: Predictive validity and longitudinal effects. Psychology and Health, 16, 391–407. 10.1080/08870440108405515

[bjhp12582-bib-0030] Hamilton, K. , van Dongen, A. , & Hagger, M. S. (2020). An extended theory of planned behavior for parent‐for‐child health behaviors: A meta‐analysis. Health Psychology, 39, 863–878. 10.1037/hea0000940 32597678

[bjhp12582-bib-0032] Inchley, J. , Currie, D. , Young, T. , Samdal, O. , Torsheim, T. , Augustson, L. , … Barnekow, V. (2016). Growing up unequal: gender and socioeconomic differences in young people’s health and well‐being. Health Behaviour in School‐Aged Children (HBSC) Study: International Report from the 2013/14 Study. World Health Organisation, Geneva.

[bjhp12582-bib-0033] Instone, R. , & Davies, E. L. (2019). Exploring the application of the Prototype Willingness Model to weight loss dieting behaviour among UK adults. Psychology, Health & Medicine, 24, 1075–1089. 10.1080/13548506.2019.1622749 31129985

[bjhp12582-bib-0034] Janssen, I. , & LeBlanc, A. G. (2010). Review systematic review of the health benefits of physical activity and fitness in school‐aged children and youth. International Journal of Behavioral Nutrition and Physical Activity, 7, 1–16. 10.1186/1479-5868-7-40 20459784 PMC2885312

[bjhp12582-bib-0035] Johnson, V. E. (2013). Revised standards for statistical evidence. Proceedings of the National Academy of Sciences, 110, 19313–19317. 10.1073/pnas.1313476110 PMC3845140 24218581

[bjhp12582-bib-0036] Kwasnicka, D. , Ntoumanis, N. , Hunt, K. , Gray, C. M. , Newton, R. U. , Gucciardi, D. F. , … Quested, E. (2020). A gender‐sensitised weight‐loss and healthy living program for men with overweight and obesity in Australian Football League settings (Aussie‐FIT): A pilot randomised controlled trial. PLoS Medicine, 17, e1003136. 10.1371/journal.pmed.1003136 32760144 PMC7410214

[bjhp12582-bib-0037] Markus, H. , & Nurius, P. (1986). Possible selves. American Psychologist, 41, 954–969. 10.1037/0003-066X.41.9.954

[bjhp12582-bib-0038] McEachan, R. R. C. , Conner, M. , Taylor, N. J. , & Lawton, R. J. (2011). Prospective prediction of health‐related behaviours with the theory of planned behaviour: A meta‐analysis. Health Psychology Review, 5, 97–144. 10.1080/17437199.2010.521684

[bjhp12582-bib-0040] Murru, E. C. , & Ginis, K. A. M. (2010). Imagining the possibilities: The effects of a possible selves intervention on self‐regulatory efficacy and exercise behavior. Journal of Sport and Exercise Psychology, 32, 537–554. 10.1123/jsep.32.4.537 20733212

[bjhp12582-bib-0041] Nurmi, J.‐E. (1991). How do adolescents see their future? A review of the development of future orientation and planning. Developmental Review, 11(1), 1–59. 10.1016/0273-2297(91)90002-6

[bjhp12582-bib-0042] Ouellette, J. A. , Gerrard, M. , Gibbons, F. X. , & Reis‐Bergan, M. (1999). Parents, peers, and prototypes: Antecedents of adolescent alcohol expectancies, alcohol consumption, and alcohol‐related life problems in rural youth. Psychology of Addictive Behaviors, 13, 183–197. 10.1037/0893-164X.13.3.183

[bjhp12582-bib-0043] Ouellette, J. A. , Hessling, R. , Gibbons, F. X. , Reis‐Bergan, M. , & Gerrard, M. (2005). Using images to increase exercise behavior: Prototypes versus possible selves. Personality and Social Psychology Bulletin, 31, 610–620. 10.1177/0146167204271589 15802656

[bjhp12582-bib-0044] Perras, M. G. M. , Strachan, S. M. , & Fortier, M. S. (2016). Possible selves and physical activity in retirees: The mediating role of identity. Research on Aging, 38, 819–841. 10.1177/0164027515606191 26408187

[bjhp12582-bib-0045] Pomery, E. A. , Gibbons, F. X. , Reis‐Bergan, M. , & Gerrard, M. (2009). From willingness to intention: Experience moderates the shift from reactive to reasoned behavior. Personality and Social Psychology Bulletin, 35, 894–908. 10.1177/0146167209335166 19429884 PMC2742327

[bjhp12582-bib-0046] Reynolds, K. J. , Lee, E. , Turner, I. , Bromhead, D. , & Subasic, E. (2017). How does school climate impact academic achievement? An examination of social identity processes. School Psychology International, 38(1), 78–97. 10.1177/0143034316682295

[bjhp12582-bib-0047] Rhodes, R. E. , & Bruijn, G. (2013). How big is the physical activity intention–behaviour gap? A meta‐analysis using the action control framework. British Journal of Health Psychology, 18, 296–309. 10.1111/bjhp.12032 23480428

[bjhp12582-bib-0048] Rhodes, R. E. , Cox, A. , & Sayar, R. (2021). What predicts the physical activity intention–behavior gap? A systematic review. Annals of Behavioral Medicine, 56(1), 1–20. 10.1093/abm/kaab044 34231844

[bjhp12582-bib-0049] Rhodes, R. E. , McEwan, D. , & Rebar, A. L. (2019). Theories of physical activity behaviour change: A history and synthesis of approaches. Psychology of Sport and Exercise, 42, 100–109. 10.1016/j.psychsport.2018.11.010

[bjhp12582-bib-0050] Rivis, A. , Sheeran, P. , & Armitage, C. J. (2006). Augmenting the theory of planned behaviour with the prototype/willingness model: Predictive validity of actor versus abstainer prototypes for adolescents’ health‐protective and health‐risk intentions. The British Journal of Health Psychology, 11, 483–500. 10.1348/135910705X70327 16870057

[bjhp12582-bib-0051] Sallis, J. F. , & Owen, N. (1998). Physical activity and behavioral medicine (Vol. 3). Thousand Oaks, CA: SAGE Publications.

[bjhp12582-bib-0052] Scott, J. J. , Morgan, P. J. , Plotnikoff, R. C. , & Lubans, D. R. (2015). Reliability and validity of a single‐item physical activity measure for adolescents. Journal of Paediatrics and Child Health, 51, 787–793. 10.1111/jpc.12836 25643749

[bjhp12582-bib-0053] Spijkerman, R. , van den Eijnden, R. J. J. M. , Vitale, S. , & Engels, R. C. M. E. (2004). Explaining adolescents’ smoking and drinking behavior: The concept of smoker and drinker prototypes in relation to variables of the theory of planned behavior. Addictive Behaviors, 29, 1615–1622. 10.1016/j.addbeh.2004.02.030 15451128

[bjhp12582-bib-0054] Sterdt, E. , Liersch, S. , & Walter, U. (2014). Correlates of physical activity of children and adolescents: A systematic review of reviews. Health Education Journal, 73(1), 72–89. 10.1177/0017896912469578

[bjhp12582-bib-0055] Strachan, S. M. , Marcotte, M. M. E. , Giller, T. M. T. , Brunet, J. , & Schellenberg, B. J. I. (2017). An online intervention to increase physical activity: Self‐regulatory possible selves and the moderating role of task self‐efficacy. Psychology of Sport and Exercise, 31, 158–165. 10.1016/j.psychsport.2016.05.001

[bjhp12582-bib-0056] Tajfel, H. (1982). Social psychology of intergroup relations. Annual Review of Psychology, 33(1), 1–39. 10.1146/annurev.ps.33.020182.000245

[bjhp12582-bib-0057] Taylor, C. (2017). The reliability of free school meal eligibility as a measure of socio‐economic disadvantage: Evidence from the millennium cohort study in wales. British Journal of Educational Studies, 66(1), 29–51. 10.1080/00071005.2017.1330464

[bjhp12582-bib-0058] Todd, J. , Kothe, E. , Mullan, B. , & Monds, L. (2014). Reasoned versus reactive prediction of behaviour: A meta‐analysis of the prototype willingness model. Health Psychology Review, 10(1), 1–24. 10.1080/17437199.2014.922895 26824678

[bjhp12582-bib-0059] Todd, J. , Kothe, E. , Mullan, B. , & Monds, L. (2016). Reasoned versus reactive prediction of behaviour: A meta‐analysis of the prototype willingness model. Health Psychology Review, 10(1), 1–24. 10.1080/17437199.2014.922895 26824678

[bjhp12582-bib-0060] Todd, J. , & van Lettow, B. (2016). A closer look at prototypes: Similarity, favourability, and the prototype willingness model. A response to the commentary of Gibbons and Gerrard. Health Psychology Review, 10(1), 47–49. 10.1080/17437199.2016.1138872 26732816

[bjhp12582-bib-0062] van Lettow, B. , de Vries, H. , Burdorf, A. , & van Empelen, P. (2016). Quantifying the strength of the associations of prototype perceptions with behaviour, behavioural willingness and intentions: A meta‐analysis. Health Psychology Review, 10(1), 25–43. 10.1080/17437199.2014.941997 25166958

[bjhp12582-bib-0063] Wassenaar, T. M. , Wheatley, C. M. , Beale, N. , Salvan, P. , Meaney, A. , Possee, J. B. , … Johansen‐Berg, H. (2019). Effects of a programme of vigorous physical activity during secondary school physical education on academic performance, fitness, cognition, mental health and the brain of adolescents (Fit to Study): Study protocol for a cluster‐randomised trial. Trials, 20(1), 189. 10.1186/s13063-019-3279-6 30940164 PMC6444886

[bjhp12582-bib-0064] Wheatley, C. M. , Davies, E. L. , & Dawes, H. (2017). Unspoken playground rules discourage adolescent physical activity in school: A focus group study of constructs in the prototype willingness model. Qualitative Health Research, 28, 1049732317744534. 10.1177/1049732317744534 29199530

[bjhp12582-bib-0065] Wheatley, C. , Johansen‐Berg, H. , Dawes, H. , & Davies, E. (2020). Perceptions of active and inactive prototypes are associated with objective measures of physical activity in adolescents. Psychology, Health & Medicine, 25, 1216–1227. 10.1080/13548506.2020.1738018 PMC7616925 32195596

[bjhp12582-bib-0066] Whitaker, L. , Long, J. , Petróczi, A. , & Backhouse, S. H. (2014). Using the prototype willingness model to predict doping in sport. Scandinavian Journal of Medicine & Science in Sports, 24, e398–e405. 10.1111/sms.12148 25371934

[bjhp12582-bib-0067] World Health Organization . (2016). Global recommendations on physical activity for health. Geneva: World Health Organization.

