# Supplementary material for: The importance of prototype similarity for physical activity: Cross‐sectional and longitudinal associations in a large sample of young adolescents
Source: Br J Health Psychol. 2022 Feb 3;27(3):915–34. doi: 10.1111/bjhp.12582 (PMC9540821; doi:10.1111/bjhp.12582)
Supplement: Supplementary file 1 — Figure S1. Flow chart showing Fit to Study sample at baseline and follow‐up. [file BJHP-27-915-s003.docx]

**Additional File 1**

**Fig. 1:** **Flow chart showing Fit to Study sample at baseline and follow-up**

Table 1.
