# Supplementary material for: The importance of prototype similarity for physical activity: Cross‐sectional and longitudinal associations in a large sample of young adolescents
Source: Br J Health Psychol. 2022 Feb 3;27(3):915–34. doi: 10.1111/bjhp.12582 (PMC9540821; doi:10.1111/bjhp.12582)
Supplement: Supplementary file 2 — Table S1. Associations between model variables and physical activity, and variance explained, in control sample only at baseline (n = 4,965) and follow‐up (n = 2,773). [file BJHP-27-915-s002.docx]

**Additional File 2**

**Table 1: Associations between model variables and physical activity, and variance explained, in control sample only at baseline (n = 4,965) and follow-up (n= 2,773)**

| **Predictor variables** | **Baseline: cross-sectional analysis** | | | | **Follow-up: longitudinal analysis** | | | |
| --- | --- | --- | --- | --- | --- | --- | --- | --- |
|  | ***R^2^*** | **ß** | **2.5% CI** | **97.5% CI** | ***R^2^*** | **ß** | **2.5% CI** | **97.5% CI** |
| **Step 1** | 0.377 |  |  |  | 0.124 |  |  |  |
| Habitual activity |  | 0.82** | 0.79 | 0.85 |  | 0.49** | 0.44 | 0.54 |
| Sex^a^ |  | -0.27** | -0.36 | -0.18 |  | -0.01 | -0.16 | 0.14 |
| eFSM^b^ |  | -0.07 | -0.19 | 0.05 |  | -0.02 | -0.23 | 0.20 |
|  |  |  |  |  |  |  |  |  |
| **Step 2** | 0.425 |  |  |  | 0.170 |  |  |  |
| Habitual activity |  | 0.53** | 0.49 | 0.57 |  | 0.32** | 0.27 | 0.38 |
| Sex^a^ |  | -0.21** | -0.30 | -0.12 |  | -0.03 | -0.17 | 0.12 |
| eFSM^b^ |  | -0.01 | -0.13 | 0.11 |  | -0.01 | -0.22 | 0.20 |
| Intention |  | 0.18** | 0.15 | 0.22 |  | 0.16** | 0.11 | 0.22 |
| Attitude |  | 0.11** | 0.07 | 0.16 |  | 0.14** | 0.08 | 0.21 |
| Subjective Norms |  | 0.10** | 0.06 | 0.14 |  | 0.04 | -0.02 | 0.10 |
| PBC: capacity |  | 0.11** | 0.07 | 0.15 |  | 0.11** | 0.06 | 0.16 |
| PBC: autonomy |  | 0.00 | -0.03 | 0.03 |  | 0.02 | -0.02 | 0.06 |
|  |  |  |  |  |  |  |  |  |
| **Step 3** | 0.438 |  |  |  | 0.182 |  |  |  |
| Habitual activity |  | 0.47** | 0.43 | 0.51 |  | 0.29** | 0.23 | 0.34 |
| Sex^a^ |  | -0.21** | -0.30 | -0.12 |  | -0.02 | -0.17 | 0.13 |
| eFSM^b^ |  | 0.00 | -0.12 | 0.12 |  | 0.01 | -0.20 | 0.22 |
| Intention |  | 0.15** | 0.11 | 0.19 |  | 0.14** | 0.09 | 0.19 |
| Attitude |  | 0.06** | 0.01 | 0.10 |  | 0.12** | 0.06 | 0.19 |
| Subjective Norms |  | 0.09** | 0.05 | 0.13 |  | 0.03 | -0.03 | 0.09 |
| PBC: capacity |  | 0.08** | 0.04 | 0.12 |  | 0.09** | 0.04 | 0.15 |
| PBC: autonomy |  | 0.00 | -0.03 | 0.03 |  | 0.02 | -0.02 | 0.06 |
| Active favourable |  | 0.05 | 0.01 | 0.09 |  | -0.00 | -0.06 | 0.05 |
| Active similar |  | 0.11** | 0.07 | 0.14 |  | 0.13** | 0.06 | 0.20 |
| Inactive favourable |  | 0.01 | -0.02 | 0.04 |  | -0.01 | -0.05 | 0.03 |
| Inactive similar |  | -0.07** | -0.10 | -0.04 |  | -0.04 | -0.08 | -0.00 |
| Willingness |  | 0.07** | 0.03 | 0.11 |  | 0.03 | -0.01 | 0.09 |

*p<0.005; **p<0.001

Fully-adjusted multilevel model including covariates of age, sex, eFSM, term/place of measurement and school effects, with confidence intervals bootstrapped. ^a^Reference category: male ^b^Reference category: not eligible for FSM
