# Supplementary figures and images for: The importance of prototype similarity for physical activity: Cross‐sectional and longitudinal associations in a large sample of young adolescents
Source: Br J Health Psychol. 2022 Feb 3;27(3):915–34. doi: 10.1111/bjhp.12582 (PMC9540821; doi:10.1111/bjhp.12582)

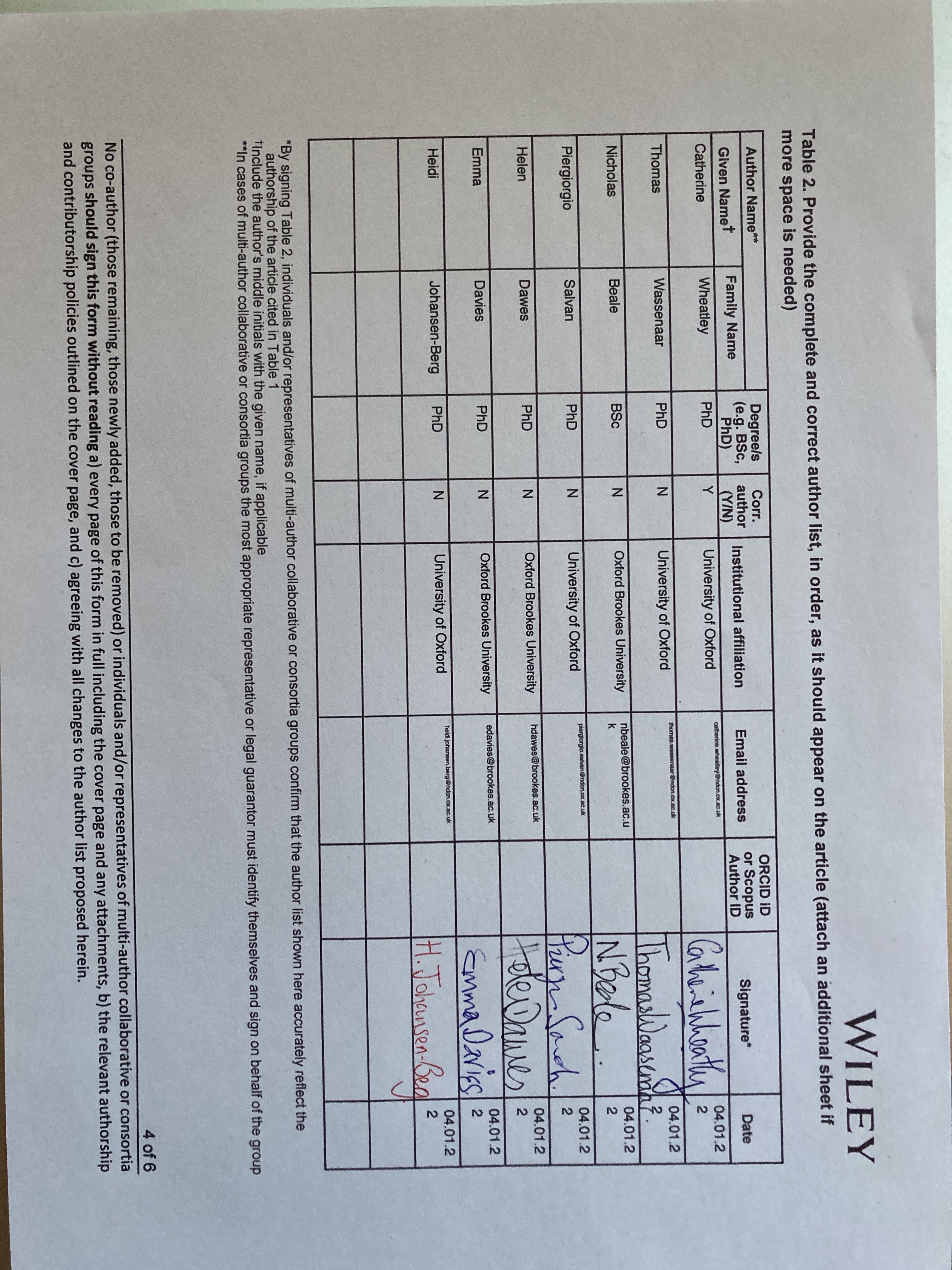

Supplement: Supplementary file 3 — Table S2. Provide the complete and correct author list, in order, as it should be appear on the article (attach and additional sheet if more space is needed). [file BJHP-27-915-s004.jpg]

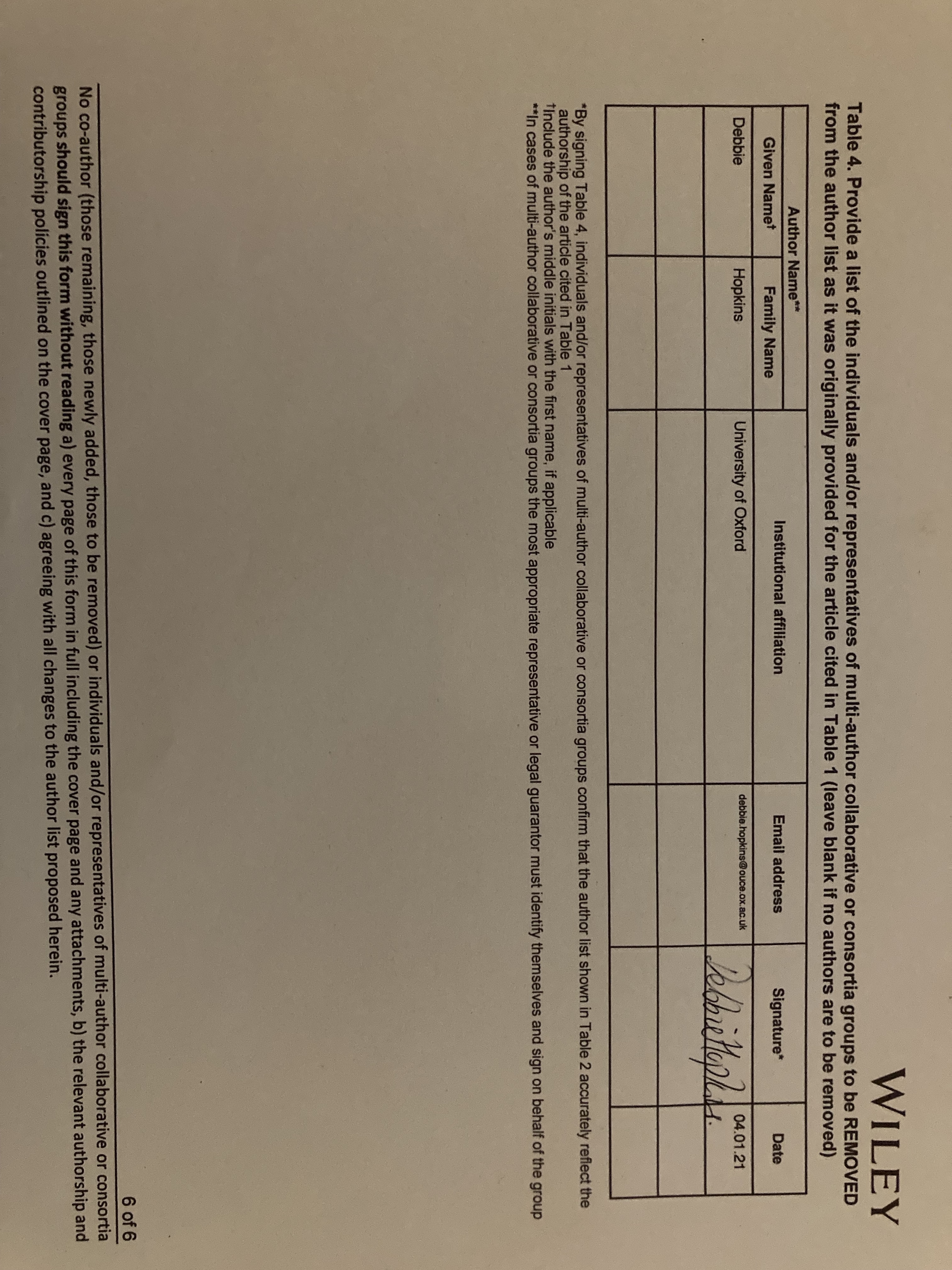

Supplement: Supplementary file 4 — Table S4. Provide a list of the individuals and/or representatives of multi‐author collaborative or consortia group to be REMOVED from the author list as it was originally provided for the article cited in Table 1. [file BJHP-27-915-s001.jpg]
